# Extracellular Vesicles, Ageing, and Therapeutic Interventions

**DOI:** 10.3390/cells7080110

**Published:** 2018-08-18

**Authors:** Nikolaos Panagiotou, Ognian Neytchev, Colin Selman, Paul G. Shiels

**Affiliations:** 1 Wolfson Wohl Cancer Research Centre, College of Medical, Veterinary & Life Sciences, Institute of Cancer Sciences, University of Glasgow, Glasgow G61 1QH, UK; n.panagiotou.1@research.gla.ac.uk (N.P.); o.neytchev.1@research.gla.ac.uk (O.N.); 2 College of Medical, Veterinary & Life Sciences, Institute of Biodiversity, Animal Health and Comparative Medicine, University of Glasgow, Graham Kerr, Glasgow G12 8QQ, UK; Colin.Selman@glasgow.ac.uk

**Keywords:** ageing, extracellular vesicles, microvesicles, exosomes, stem cells, epigenetics

## Abstract

A more comprehensive understanding of the human ageing process is required to help mitigate the increasing burden of age-related morbidities in a rapidly growing global demographic of elderly individuals. One exciting novel strategy that has emerged to intervene involves the use of extracellular vesicles to engender tissue regeneration. Specifically, this employs their molecular payloads to confer changes in the epigenetic landscape of ageing cells and ameliorate the loss of functional capacity. Understanding the biology of extracellular vesicles and the specific roles they play during normative ageing will allow for the development of novel cell-free therapeutic interventions. Hence, the purpose of this review is to summarise the current understanding of the mechanisms that drive ageing, critically explore how extracellular vesicles affect ageing processes and discuss their therapeutic potential to mitigate the effects of age-associated morbidities and improve the human health span.

## 1. Introduction

There is a growing number of aged individuals in the global population. It is estimated that within the next few years, people aged 65 and older will outnumber children below the age of 5 for the first time in our history [1]. It is predicted that this demographic shift will inevitably be accompanied by a significant increase in the incidence of age-associated morbidities, including cardiovascular disease, chronic kidney disease, diabetes, neurodegeneration, osteoarthritis, osteoporosis, and cancer, among many others. Consequently, this rise of age-related disorders will financially burden health care and social security systems around the world. The global economic burden of health care is estimated at €35 trillion over the next two decades, accounting for 15% of the costs of social security systems and for 20% of the costs of health care systems in the European Union (EU) [2]. There is, thus, a vital need to improve our understanding of the mechanisms underpinning ageing processes and to develop novel therapeutic strategies in order to mitigate the effects of age-related morbidities. The upshot will be an improvement in health span (i.e., more years of healthy living) and a reduction in age-related co-morbid conditions.

## 2. Ageing

**Figure 1:**
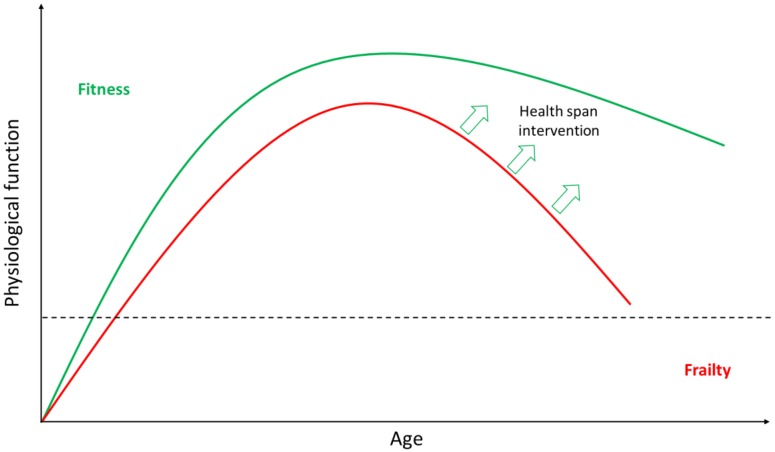
Schematic representation of different trajectories of ageing. The green line indicates a good trajectory for normative ageing. The red line indicates a poorer ageing trajectory, associated with a steeper decline in age-related physiological functional capacity. Loss of functional capacity results in fitness decline, frailty, and a greater risk of mortality. Improving the human health span would require interventions that are capable of pushing the poorer trajectory of ageing up and to the right, providing more years of good health and vitality.

Ageing, either at the organismal or cellular level, can be defined by the loss of physiological function over time (Figure 1). Ageing is not a passive process [3] but one actively regulated by distinct molecular pathways, layered below a loss of functional physiological capacity and correlated with frailty and increased likelihood of death. The underpinning mechanisms are considered to be evolutionarily conserved across taxa [4,5] and reflected in nine distinct hallmarks [6]. These comprise genomic instability, telomere shortening, epigenetic alterations, loss of proteostasis, deregulated nutrient sensing, mitochondrial dysfunction, senescence, stem cell exhaustion, and altered intercellular communication [6]. These features mirror the acquisition of allostatic load, which reflects the wear and tear on the organism, and eventual allostatic overload [7], correlated with dysregulated physiological function with ageing [8,9,10,11,12]. Consequently, they reflect the accumulation of cell and tissue damage, the loss of repair capacity, and an increased probability of neo-oncogenesis due to dysregulated cell proliferation [13,14,15].

### 2.1. Ageing is Highly Heterogeneous

It is important to recognise that not all individuals age at the same rate or in the same way [16]. Indeed, individual organ systems, cells, organelles, and molecules within individuals may age at significantly different rates [2]. In order to explain the inter-individual and intra-individual variations observed during normative ageing, we need to further understand how extrinsic factors, including environmental, physical, psychological, and socioeconomic factors, interplay with intrinsic elements, such as genetics and epigenetics. These factors may act synergistically, independently, or cumulatively. How they do so is still the focus of ongoing research. Recent studies focusing on the epigenetics of human ageing and how epigenetic changes can significantly impact the way we age [17,18,19,20,21] have provided novel insight into a series of epigenetic modifications that influence the functional decline that is associated with organismal ageing [22,23,24,25,26]. However, exacly how these epigenetic changes are influenced by extrinsic factors among different individuals and different organ systems has not yet been determined. Thus, age-related morbidities and their relationship with psychosocial, nutritional, and lifestyle factors, as well as with genetic and epigenetic determinants of ageing, remain to be elucidated [17,27].

In this respect, the study of animals and how they mitigate some of the loss of physiological function, common to ageing and diseases of ageing in humans, may be of benefit. Interestingly, almost all of the diseases that affect humans have an equivalent in the animal kingdom [28]. Hence, the success of such an approach depends on a study of biomimicry as opposed to simply comparative biology. For instance, studying the long-lived naked mole rats (*Heterocephalus glaber*) and how they are protected from oxidative stress and cancer, could assist in the development of strategies that prevent or slow down premature ageing. Additionally, the study of rougheye rockfish (*Sebastes aleutianus*) and bowhead whale (*Balaena mysticetus*), which both have documented lifespans of >200 years, could potentially enhance our understanding of autonomous and non-autonomous cellular senescence during ageing [28].

Despite advances in geroscience, which have allowed us to better understand how we age, there is still a pressing need to better understand the mechanisms that drive ageing throughout the life course and not just in the latter half of life.

### 2.2. Anti-Ageing Strategies

A number of strategies appear capable of mitigating against the effects of age-related morbidities, with lifestyle factors such as nutrition, exercise, and other psychosocial factors impacting positively on health and ageing [29]. The transplantation of organs and tissues can also improve outcomes for patients with age-associated organ failure. Moreover, while stem cell-based therapies hold significant promise, a number of hurdles remain to be overcome before they can be of common clinical useage. These include issues concerning safety, cancer risk, proper cell selection and differentiation, delivery to the target site, cost, and scalability [30]. Therefore, cell-free therapeutics capable of activating a repair process would be a suitable alternative to stem cell transplantation.

One novel approach that has been developed recently exploits the use of extracellular vesicle biology. Alterations in intercellular communication have already been identified as one of the hallmarks of ageing [6] and extracellular vesicles act as paracrine mediators of such intercellular communication. However, their role remains largely unexplored in the context of ageing, and this review aims to discuss recent advances in the field. Interestingly, extracellular vesicles have been associated both with physiological and pathological ageing and thus offer significant diagnostic potential [7,31]. Additionally, the potential of stem cell-derived extracellular vesicles has also been explored as a novel regenerative strategy to combat age-related diseases [32]. Indeed, extracellular vesicles offer a cell-free therapeutic alternative to stem cell transplantation, avoiding the associated risks. Consequently, the study of extracellular vesicles may prove helpful in obtaining a more comprehensive picture of ageing processes, mitigating any associated adverse effects on health span and understanding the mechanisms underpinning the spread of allostatic load across the human body.

## 3. Extracellular Vesicles

**Figure 2:**
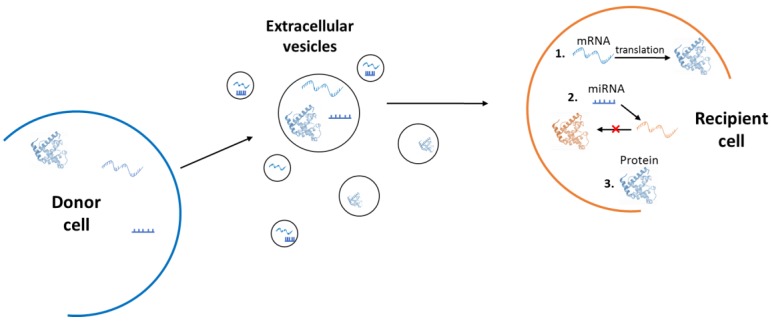
Extracellular vesicle mechanisms of action. Extracellular vesicles can confer functional changes and alter the phenotype of recipient cells through the action of their molecular payload. This involves: 1. extracellular vesicle mRNA translation within the recipient cell cytoplasm, 2. regulation of gene expression mediated by extravesicular miRNA, and 3. direct extracellular vesicle protein action.

A component of physiological homeostasis has been attributed to the action of extracellular vesicles, which have proven roles in tissue maintenance and repair [32]. Extracellular vesicles comprise of all membranous vesicles that are secreted by cells and that encapsulate bioactive molecules, such as proteins and nucleic acids [32]. These vesicles have been recognised as important mediators of cell-to-cell communication, both in physiological and pathological states [31]. Extracellular vesicles can be secreted from almost all cell types and are found in most human biological fluids, including plasma, urine, synovial fluid, and bronchial lavage fluid [33,34]. They are capable of affecting the phenotype of recipient cells, after local or systemic circulation, by intercellular transfer of their molecular payloads [35,36]. Their bioactive cargo can include double-stranded DNA [37], mRNA, micro-RNAs (miRNAs) and other non-coding RNAs [37,38,39,40]. We have previously proposed at least three mechanisms of action (Figure 2) through which the extracellular vesicles can alter the phenotype of recipient cells [32]. Firstly, through the translation of extravesicular mRNAs. Once extracellular vesicle endocytosis has taken place and the mRNAs of extracellular vesicle origin have been released within the recipient cell cytoplasm, their translation can take place. The translated proteins can then directly affect the phenotype of the cell. Secondly, through miRNA-mediated regulation of the host cell mRNAs at the post-transcriptional level. Thirdly, through direct extravesicular protein action within the recipient cells [32]. Transfer of these biologically active cargoes can generate functional changes directly in recipient cells and alter their epigenetic landscape. These may also provide a molecular basis for the effects of non-cell autonomous senescence and the spread of allostatic load across the body [7,38].

### 3.1. Microvesicles and Exosomes

The majority of previous research has focused on two distinct classes of extracellular vesicles: exosomes and microvesicles. Exosomes have a size range of 30–100 nm diameter and are released via multi-vesicular endosome fusion with the plasma membrane [41,42,43,44,45]. Microvesicles are bigger than exosomes, ranging in size from 100 to 1000 nm, and are shed directly from the plasma membrane [43,45]. Apart from differences in size and point of origin, these two broad classes of vesicles can be distinguished by their membrane proteins and RNA markers [46]. For instance, exosomes are positive for CD9 marker expression, while microvesicles do not express CD9 on their surface. Moreover, the CD40 membrane protein is present only on microvesicles and not exosomes [47].

### 3.2. Isolation Methodology

Extracellular vesicles can be isolated from the cell culture media of numerous cell types or from body fluids primarily via centrifugation and ultrafiltration, while other isolation techniques have been critically reviewed elsewhere [48,49]. Brief mention of methodology is important, as centrifugation, while enabling isolation of the correct size class of vesicles, often results in trappage of other vesicle types [50,51]. Ultrafiltration, combined with characterisation of vesicle membrane proteins, is required to subsequently guarantee successful purification from other extracellular vesicle contaminants and a successful vesicle preparation of the desired type. This is critical to ensure and validate the correct separation between the different size classes of extracellular vesicles in order to study their role in ageing, as well as their therapeutic potential.

## 4. Role of Extracellular Vesicles in Ageing

Extracellular vesicles are a heterogeneous population whose molecular payloads have been shown to be dependent on the cell type of origin. Most notably, stem cell and cancer cell extracellular vesicles have been identified to have a distinct composition [52,53]. These findings could allow for more accurate identification of extracellular vesicle subtypes and pave the way for the use of extracellular vesicles as biomarkers of diagnostic value. Indeed, extracellular vesicles have recently been under investigation as possible biomarkers of both cancer and ageing [53,54,55]. Extracellular vesicles secreted by cancer cells can provide information on driver mutations, molecular subtypes, or drug resistance [54], while miRNAs from extracellular vesicles derived from saliva or serum have shown good association with age [55,56].

### 4.1. A Double-Edged Sword

Extracellular vesicles have been identified as key mediators of paracrine signaling, both in physiological and pathological states associated with ageing [57]. They can mediate beneficial effects, in processes such as wound healing and tissue regeneration, as well as detrimental effects, such as promoting oncogenesis and cellular senescence [58]. Additionally, extracellular vesicles may have a range of age-related effects including promotion of oncogenic transformation, resistance to apotosis, epithelial-to-mesenchymal transition and metastasis, stimulation of angiogenesis, evasion of immunosurveillance, and resistance to anticancer treatment [40,52,58,59,60,61,62,63]. These negative properties, however, can be exploited for therapeutic benefit, as they are also capable of spreading sensitivity to treatment across a population of cancer cells [52].

Exosomes can mediate the removal of cytotoxins from cells. For instance, as a branch of the endolysosomal pathway, they can contribute to cellular homeostasis by removing misfolded or unwanted proteins and cytoplasmic DNA, both of which can be targeted to multivesicular bodies and released via exocytosis [57,64]. Thus, exosomes potentially play a key role in the age-related loss of proteostasis [65]. Conversely, microvesicles have been demonstrated to have a direct role in enabling tissue regeneration by delivering bioactive molecules to target cells [47]. One negative connotation for exosomes and the miRNAs they contain, is that they may provide a mechanism responsible for the spread of allostatic load between different tissues and organs across the body, thus modulating the rate of ageing at a systemic level [7]. Similarly, microvesicles released by cancer cells may facilitate cancer progression by transferring payloads that stimulate invasion, metastasis, and angiogenesis or by removing chemotherapeutic drugs from cancerous cells [66].

### 4.2. Role in Cellular Senescence

Numerous studies have suggested that endogenous or exogenous stress can affect the amount or content of secreted extracellular vesicles [7,38,58,67]. It has been speculated that this can provide a communication channel through which cells can send a “distress” signal, enabling other cells to better prepare or react to the stressful stimulus [58]. While this process can have beneficial effects in the short term, it can also become maladaptive and constitute one of the paths through which stress can accelerate the rate of ageing [7]. For instance, senescence is thought to be a protective mechanism that prevents damaged cells from undergoing malignant transformation. It has also been implicated in both developmental processes and wound healing [68,69,70]. Senescent cells release an increased amount of extracellular vesicles, which may be a non-canonical part of the senescence-associated secretory phenotype (SASP) [67,71,72]. Through the SASP, senescent cells can spread the senescent phenotype to neighbouring non-damaged cells via a bystander effect [73]. Given the accumulation of senescent cells in tissues as part of normative ageing, extracellular vesicles may thus contribute to the creation of a pro-inflammatory and pro-senescent microenvironment that acts as a significant contributor to physiological ageing and age-related disease [74]. Indeed, senescence-associated extracellular vesicles appear to be a novel SASP factor involved in age-associated lung disease. For instance, elevated levels of exosomal miR-21 in the serum have been associated with idiopathic pulmonary fibrosis [75]. The exosomal miR-21 might thus play a significant role as a SASP factor. Cellular senescence is a key area of biogerontology research. The potential therapeutic effect of ablating senescent cells in vivo, through genetic or pharmacological means, has already been demonstrated in pre-clinical models in mammals [8,69]. Benefits from the clearance of senescent cells in a number of models have included protection from cancer, cardiovascular disease, and a lifespan increase of up to 30% [8,9,69,76].

### 4.3. Role in Genomic Instability

Extracellular vesicles may also play a role in promoting another hallmark of ageing, namely increasing genomic instability, through the transfer of retrotransposons [77]. Extracellular vesicles derived from cancer cells have in particular been shown to be highly enriched in retrotransposon sequences, which they can transfer to recipient cells [77]. Retrotransposons are mobile DNA elements capable of creating and inserting multiple copies of themselves into the host genome [78]. Indeed, around half of mammalian genomes are composed of such repetitive sequences [79]. It is worth noting that the process of retrotransposition involves a double-strand break in the DNA and therefore even an unsuccessful transposition event (i.e., one that does not end with an insertion of the retrotransposon sequence) can still be mutagenic [78]. Intriguingly, retrotransposon expression has been found to increase in senescent cells and may be a driver of ageing [78].

### 4.4. Role in the Spread of Disease

Extracellular vesicle secretion has also been shown to change with age and disease. For instance, aged rat mesenchymal stem cells (MSCs) secrete more exosomes than their young counterparts. However, these vesicles have a lower content of anti-inflammatory miRNAs [52] and thus could theoretically contribute to the inflammatory burden that comes with age. In a separate study, MSC microvesicles that originated from older rats were demonstrated to have a lower content of miR-133b-3p and miR-294, two miRNAs that inhibit TGF-β1-mediated epithelial-to-mesenchymal transition, which is a process that contributes to renal fibrosis [80]. Thus, microvesicles from aged MSCs may be less capable of protecting kidneys from fibrosis, which in turn may lead to the development of chronic kidney disease, a proven disease of accelerated ageing [27].

Extracellular vesicles have also been implicated in the spread of a number of other diseases, for instance by shuttling toxic protein aggregates or misfolded proteins, including those involved in Alzheimer’s disease and prion diseases [7,58,60,81]. In addition, increased exosome secretion by the retinal pigmented epithelium may be a factor contributing to the accumulation of the extracellular material drusen, which is observed in age-related macular degeneration [58,82]. Exosomes have been implicated in the transformation of vascular smooth muscle cells (VSMCs), which contributes to vascular calcification [83]. Exosomes secreted by VSMCs exposed to stressful stimuli promote local calcification, while blocking exosome secretion prevents calcification [83,84]. Extracellular vesicles are also able to spread a variety of pathogens including HIV, hepatitis C, and *Toxoplasma*, among others [81]. Finally, extracellular vesicles have also been identified to aggravate autoimmune diseases such as rheumatoid arthritis [81].

Overall, extracellular vesicles have been implicated both in physiological and pathological ageing, and it is essential that we further comprehend their biology. Additionally, it is important to distinguish and understand the roles that different extracellular vesicle subclasses play during ageing.

## 5. Combating Diseases of Ageing with Extracellular Vesicles

**Figure 3:**
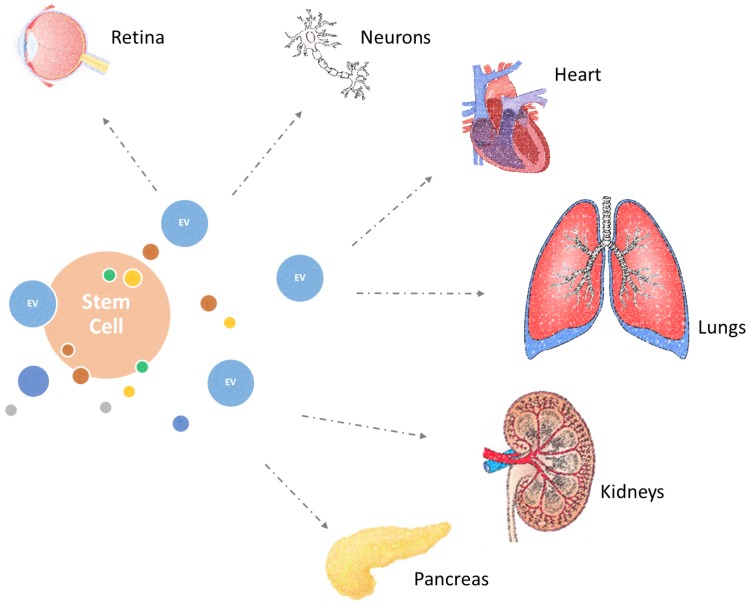
Extracellular vesicles (EV) and their therapeutic applications. Ageing results in loss of functional capacity in a number of key organs. Microvesicles, derived from stem cells or stem cell regulatory cell types, such as Pathfinder cells, can become a novel cell-free therapeutic intervention to mitigate the undesirable effects of ageing by enhancing tissue regeneration and thus improving the human health span.

Although once thought to be cell debris, there is now solid evidence to indicate that extracellular vesicles play a vital role in intercellular communication, stem cell regulation, and critically tissue regeneration [85,86,87,88]. Specifically, extracellular vesicles, such as microvesicles and exosomes, isolated from stem cells or stem cell regulatory cell types, have been revealed to display significant regenerative properties in a number of cases of age-associated tissue loss of function [32]. Through the transfer of bioactive molecules, these extracellular vesicles are capable of conferring therapeutic effects in cases of cardiac, lung, retinal, neural, pancreatic, and kidney damage, which accompany ageing (Figure 3). Hence, there is growing interest in exploring extracellular vesicles as therapeutic mediators capable of conferring beneficial changes in the epigenetic landscape of ageing cells and organs.

### 5.1. Cardioprotective Effects

Human induced pluripotent stem cells (iPSCs) have been used as a source of microvesicles for therapeutic applications. These iPSC-derived microvesicles were able to confer iPSC regenerative attributes to adult somatic cells, through the transfer of their molecular payloads, comprising a series of RNAs, miRNAs, and proteins. As a result, the iPSC-derived microvesicles exerted proliferative and protective effects on cardiac MSCs, while also enhancing their differentiation potential, by impacting on their transcriptome and proteomic profile. These microvesicles were also capable of transferring exogenous transcripts from genetically modified iPSCs. Hence, future therapeutic strategies can benefit from the genetic enhancement of the content of extracellular vesicles [89]. These observations were also confirmed in vivo, where both microvesicles and exosomes, isolated from iPSCs, were able to transfer miRNAs to the ischemic myocardium of an acute myocardial ischemia/reperfusion injury mouse model and protect the cardiomyocytes [90]. Additionally, exosomes of MSC origin were involved in the maintenance of tissue homeostasis, protecting ischemic cardiomyocytes by transmission of miR-22, leading to reduced apoptosis. The miR-22 anti-apoptotic effect was mediated by direct targeting and subsequent downregulation of the methyl CpG binding protein 2, which controls gene expression through epigenetic regulation [91].

### 5.2. Treatment of Lung Diseases

Moreover, the therapeutic potential of MSCs and their extracellular vesicles has been investigated in vitro for the treatment of acute lung injury and acute respiratory distress syndrome- [92,93,94]. The significance of paracrine factors for the treatment of the above disorders was demonstrated in an acute lung injury model of rat alveolar epithelial cells through restoration of sodium transport and preservation of epithelial permeability [95]. MSC-derived exosomes were also able to provide a protective effect in a model of hypoxia-induced pulmonary hypertension [96]. Similarly, microvesicles originating from MSCs have been demonstrated to provide significant therapeutic efficacy in the presence of acute lung injury and acute respiratory distress syndrome in mice [97].

### 5.3. Regeneration of the Retina

Microvesicles derived from mouse embryonic stem cells have also been explored as a novel therapeutic intervention for the regeneration of the retina. The microvesicles can transfer RNAs and miRNAs to Müller cells, inducing pluripotency and allowing them to differentiate into cells of retinal lineage [98].

### 5.4. Neural Regeneration

The therapeutic potential of microvesicles and exosomes for the development of interventions that can treat damage in the nervous system has also been discussed elsewhere [99]. Indeed, there is strong supporting evidence to suggest that the MSC-based therapies, applied in numerous models of neurodegenerative disorders, confer regenerative effects via paracrine mechanisms, involving extracellular vesicles, such as microvesicles and exosomes [100]. Interestingly, miR-133b transfer by MSC exosomes demonstrates beneficial effects on neural cells in rodent stroke models [101,102,103]. Furthermore, microvesicles isolated from macrophages have been shown to confer regeneration to peripheral nerves, stimulate proliferation and migration of Schwann cells, and contribute to axonal regeneration after nerve injury [104]. However, the potential of extracellular vesicles to transfer toxic molecules, as may occur in neurodegenerative diseases such as Alzheimer’s and Parkinson’s disease, necessitates that further investigation is required in order to develop safe and effective strategies to mitigate against neuronal dysfunction [105].

### 5.5. Treatment of Renal and Pancreatic Damage

In the instance of renal recovery following damage or age-related dysfunction, microvesicles of MSC origin can be protective following glycerol-induced, ischemia-reperfusion and cisplatin-induced acute kidney injury. This effect has been particularly attributed to the horizontal transfer of mRNA and miRNA molecules [106,107,108]. MSC microvesicles can also reverse acute and chronic kidney injury through the inhibition of apoptosis and subsequent stimulation of proliferation [109,110]. This is congruent with seminal data derived from the use of Pathfinder cells. These cells, which are a putative stem cell regulatory cell type, were able to enhance the repair of kidneys and pancreata in mice, following acute ischemic renal damage and streptozotocin (SZT)-induced diabetes, respectively [111,112]. This therapeutic effect was determined to be paracrine in nature, involving the action of microvesicles that were capable of working across a species barrier in a concordant xenotranspalantation model. Interestingly, only the administration of microvesicles, but not exosomes, was able to mediate the functional recovery of the pancreas in this model [47]. Critically, these observations were incongruent with previous observation using exosomes in ischemia models in mice but may be attributable to exosome preparations in these other models being contaminated with microvesicles. In support of such an assertion, microvesicles from endothelial progenitor cells have demonstrated therapeutic potential in the treatment of kidney damage following ischemia-reperfusion injury in rats. It has also been suggested that this effect was miRNA-mediated and involved miRNA delivery to resident renal cells [113].

### 5.6. Systemic Benefits

Finally, extracellular vesicles have been implicated in mediating the systemic benefits of exercise. Following exercise, muscle and other tissues increase the release of microvesicles and exosomes into the circulation, transferring a series of factors, termed exerkines [114]. They include anti-inflamatory peptides, RNA species, and metabolites, which are responsible for facilitating peripheral organ cross talk. It has been proposed that extracellular vesicles following exercise or bioengineered extracellular vesicles that incorporate therapeutic exerkines, could have application in the tratment of obesity, type 2 diabetes melitus, atherosclerosis, and other age-associated metabolic disorders [114,115].

## 6. Conclusions

Studying the role of extracellular vesicles, in cell-to-cell communication and in the context of physiological and pathological ageing, will enhance our understanding of the ageing process and the mechanisms underpinning the spread of allostatic load across the human body. Additionally, understanding the associated mechanisms of action, during maintenance of organismal homeostasis and tissue regeneration throughout the life course, could prove beneficial for the development of novel cell-free therapeutic interventions that could improve the health span and combat age-related loss of function and diseases.

Extracellular vesicles and specifically microvesicles are in clinical trials or moving into clinical development on a number of fronts [116]. In comparison to cell-based therapies, this cell-free regenerative strategy offers a lower risk and potentially higher scalability. Finally, production of artificial vesicles, which can carry and transfer the required bioactive payload, such as therapeutic miRNAs, to target cells could be developed as an alternative therapeutic strategy, to facilitate tissue regeneration and deal with organ loss of function and disease that comes with ageing.
